# Interaction with Soil Bacteria Affects the Growth and Amino Acid Content of *Piriformospora indica*

**DOI:** 10.3390/molecules25030572

**Published:** 2020-01-28

**Authors:** Jorge A. Leyva-Rojas, Ericsson Coy-Barrera, Rüdiger Hampp

**Affiliations:** 1 Faculty of Basic and Biomedical Science, Universidad Simón Bolivar, Barranquilla 080002, Colombia; 2 Institute of Microbiology and Infection Biology (IMIT), University of Tübingen, Auf der Morgenstelle 5, 72076 Tübingen, Germany; ruediger.hampp@uni-tuebingen.de; 3 Faculty of Basic and Applied Science, Universidad Militar Nueva Granada, Cajica 250247, Colombia

**Keywords:** *Piriformospora indica*, endophytic fungi, mycorrhiza helper bacteria, amino acid content

## Abstract

Exploration of the effect of soil bacteria on growth and metabolism of beneficial root endophytic fungi is relevant to promote favorable associations between microorganisms of the plant rhizosphere. Hence, the interaction between the plant-growth-promoting fungus *Piriformospora indica* and different soil bacteria was investigated. The parameters studied were fungal growth and its amino acid composition during the interaction. Fungus and bacteria were confronted in dual cultures in Petri dishes, either through agar or separated by a Perspex wall that only allowed the bacterial volatiles to be effective. Fungal growth was stimulated by *Azotobacter chroococcum*, whereas *Streptomyces anulatus* AcH 1003 inhibited it and *Streptomyces* sp. Nov AcH 505 had no effect. To analyze amino acid concentration data, targeted metabolomics was implemented under supervised analysis according to fungal-bacteria interaction and time. Orthogonal partial least squares-discriminant analysis (OPLS-DA) model clearly discriminated *P. indica*–*A. chroococcum* and *P. indica*–*S. anulatus* interactions, according to the respective score plot in comparison to the control. The most observable responses were in the glutamine and alanine size groups: While *Streptomyces* AcH 1003 increased the amount of glutamine, *A. chroococcum* decreased it. The fungal growth and the increase of alanine content might be associated with the assimilation of nitrogen in the presence of glucose as a carbon source. The *N*-fixing bacterium *A. chroococcum* should stimulate fungal amino acid metabolism via glutamine synthetase-glutamate synthase (GS-GOGAT). The data pointed to a stimulated glycolytic activity in the fungus observed by the accumulation of alanine, possibly via alanine aminotransferase. The responses toward the growth-inhibiting *Streptomyces* AcH 1003 suggest an (oxidative) stress response of the fungus.

## 1. Introduction

The establishment of the symbiosis between beneficial root endophytic and mycorrhizal fungi and plant roots can be affected by other microorganisms in the rhizosphere, especially by bacteria, so-called “mycorrhiza helper bacteria” (MHB) [1]. In the last two decades, a range of bacteria has been reported to be associated with the mycorrhizosphere or surface of the mycorrhizal structure of different host plants [2]. Studies by other authors demonstrated that the rhizosphere microbiome could have a positive, negative, or no impact on the mycorrhizal symbiosis, depending on the bacterial isolates [3,4,5,6]. Garbaye proposed five potential mechanisms underlying the MHB effect: (i) Enhancement of plant susceptibility to mycorrhizal colonization, (ii) enhancement of the root-fungus recognition process, (iii) nutritional improvement of fungal growth, (iv) a beneficial change in the composition of the rhizosphere soil, and (v) stimulation of fungal propagules germination [7]. It has been proposed that active bacterial attachment and colonization of the mycorrhizal surfaces are required for MHB to influence mycorrhizal plant growth and health [8,9]. Streptomycetes studies demonstrated that the exudation of bacterial compounds can both inhibit pathogenic fungi growth and support symbiotic fungi growth [10,11]. They can, however, also improve infection by plant-pathogenic fungi such as *Heterobasidion abietinum* [12].

*Piriformospora indica* is a root endophytic fungus, originally isolated from the Thar Desert in India [13]. It interacts beneficially with a broad spectrum of plants and is known to enhance plant growth, biomass production, drought tolerance, salt tolerance, phosphorus acquisition, and resistance to pathogens [14,15]. In fact, *P. indica* is functionally similar to arbuscular mycorrhiza fungi (AMF) except that it can grow axenically. The effect of MHB has also been observed with this plant-growth-promoting fungus in dual culture with rhizobacteria. It was found that *Azotobacter chroococcum*, *Azospirillum* spec. and *Bradyrhizobium* spec. promoted fungal growth. In contrast, *Pseudomonas fluorescens* and *Pseudomonas putida* completely blocked fungal growth [16].

There are some important impacts on fungus of such associations with soil bacteria, such as on the fungal growth but also on the fungal amino acid metabolism [17]. In this regard, during the symbiosis, the plant enters mainly to provide glucose to the fungus, and it will provide amino acids and other nutrients (e.g., phosphate) to the plant within the course of carbon flux. Therefore, describing the influence of soil bacteria on fungal amino acid metabolism is particularly important to define a parameter of functional bacteria–fungus association for plant nutrition [18].

In this study, we focused our investigation on the potential effect of soil bacteria on fungal growth and amino acid metabolism. For this purpose, co-cultures of *P. indica* with a range of bacterial isolates (*Azotobacter chroococcum*, *Pseudomonas fluorescens*, *Pseudomonas putida*, and the strains of Streptomyces AcH 1003, AcH 2009, and AcH 505) were established on Petri dishes. Here, we report that two soil bacteria affected both fungal development and amino acid metabolism.

## 2. Results

### 2.1. Dual Culture Assay of P. indica with Soil Microorganisms

**Figure 1:**
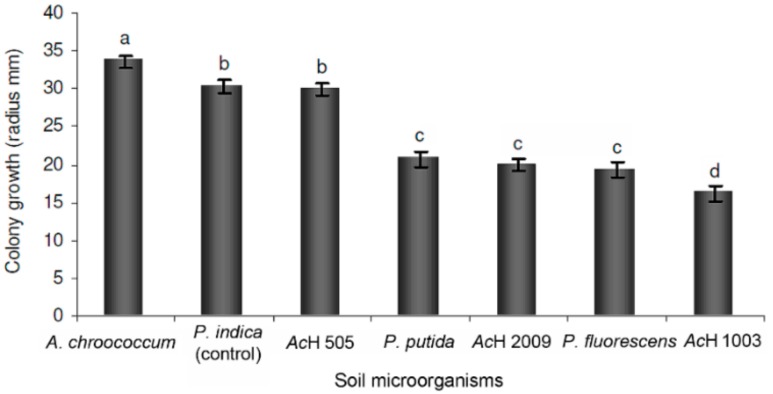
Effect of the soil microorganisms on the growth of *Piriformospora* *indica* after 12 days of dual culture. *Azotobacter chroococcum*, *Streptomyces* strains (AcH 505, AcH 2009, and AcH 1003 (*S. anulatus*)), *Pseudomonas putida* and *P. fluorescens*. Control corresponds to *P. indica* growing alone. Means with the same letters were not significantly different according to one-way ANOVA and the Tukey test (*p* < 0.05).

The influence of soil microorganisms on the growth of *Piriformospora indica* was initially determined in dual culture (DC) with the bacteria *Azotobacter chroococcum*, *Streptomyces* sp. nov 505 (AcH505), *Streptomyces* 2009 (AcH2009), *Streptomyces anulatus* 1003 (AcH1003), *Pseudomonas putida* and *Pseudomonas fluorescens*. After twelve days significant growth stimulation (*p* < 0.05) was observed in the mycelia of fungi interacting with *A. chroococcum*. In another case, growth inhibition was observed in the interactions with the two *Pseudomonas*-strains and the two *Streptomyces*-strains AcH 2009 and AcH 1003. The assay with *Streptomyces* sp. nov AcH 505 (AcH 505) showed no significant influence on fungal growth (Figure 1).

### 2.2. P. indica in Dual Culture with A. chroococcum and S. anulatus 1003

**Figure 2:**
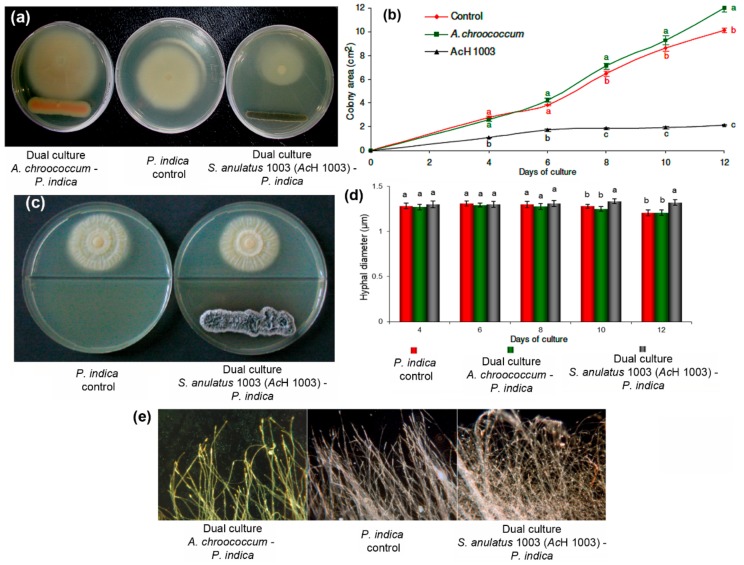
(**a**) Effect of the soil bacteria on the growth of *P. indica* after 12 days of dual culture. (**b**) Time course of *P. indica* (control) in dual cultures (DC) with *A. chroococcum* and *S. anulatus* AcH 1003. Means with the same letter were not significantly different according to one-way ANOVA and the Tukey test (*p* < 0.05). These tests were performed on growth data per recording day in order to observe differences between control and DC. (**c**) Results of the experiment to observe the plausible effect of volatile substances produced by AcH 1003 on the growth of *P. indica* after eight days of culture in split Petri-dishes. (**d**) Determination of *P. indica* hyphal thickness in DC with *A. chroococcum* and *S. anulatus* AcH 1003. Means with the same letter were not significantly different according to one-way ANOVA and the Tukey test (*p* = 0.05). (**e**) Impact of soil bacteria on *P. indica* mycelium morphology after 12 days of interaction (optical micrograph at 100×).

The soil bacteria *A. chroococcum* and *S. anulatus* 1003 (AcH 1003) were used to determine the effect of the interaction (in dual culture) on *P. indica’s* growth rate. Due to the non-isodiametric development of the colony in response to the bacteria, only the area of the colony facing the bacterial inoculum (Figure 2a) was measured. The increase of fungal growth in dual cultures was recorded from day 4 in two-day-intervals (Figure 2b). *A. chroococcum* showed a growth-promoting effect on the fungus. This effect was visible on day 6 and exhibited an important increase on day 8. On the other hand, a significant inhibitory growth effect (*p* < 0.05) of *S. anulatus* 1003 was observed from the 4th day onwards in comparison to *P. indica* control and the dual culture with *A. chroococcum*.

Parallel to the time course of growth experiments, the effect of volatile compounds produced by *S. anulatus* 1003 was also tested. For that, split Petri-dishes containing bacteria and fungi were cultivated under the same conditions as described for the time course. In this case, no effect of bacterial volatiles on fungal growth was apparently observed after eight days of co-culture (Figure 2c).

#### Morphology of *P. indica* Hyphae during Dual Culture with Soil Bacteria

Alterations in the hyphal thickness and mycelium morphology of *P. indica* in interaction with the soil bacteria were observed. In general, significant changes (*p* < 0.05) in the hyphal thickness were then observed from the 10th day during the interaction with *S. anulatus* AcH 1003, whereas no substantial changes were registered in the DC with *A. chrococcum*. In this regard, the hyphae were found to be 3%–5% thinner to than the control during interaction with *A. chroococcum*, whereas DC with *S. anulatus* AcH 1003 generated an increase above 8% in comparison to that of control (Figure 2d). In addition, changes in the mycelium morphology from the sixth day were also observed in the DC with *S. anulatus* AcH 1003, accompanied by increased hyphal density and sporulation. In contrast, the hyphae presented a slightly increased hyphal extension and reduced and delayed sporulation during interaction with *A. chroococcum* (Figure 2e).

### 2.3. Free Amino Acid Content in P. indica

**Figure 3:**
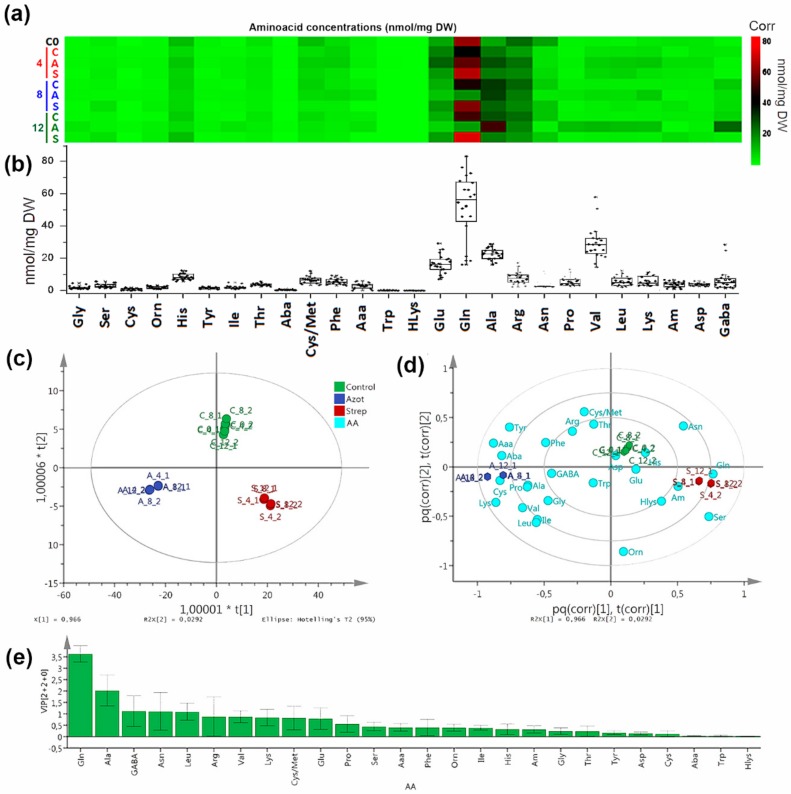
(**a**) Heatmap using ward algorithm for the variations of AA contents (expressed as nmol/mg dry weight (DW)) among interaction type (**C0** = *P. indica* control at t = 0 day, **C** = *P. indica* control, **A** = *P. indica*+*A.chroococcum*, **S** = *P. indica* + *S. anulatus* 1003 (AcH 1003)) and time (in days) of interaction (**4** = 4 days, **8** = 8 days, **12** = 12 days). (**b**) Boxplot for AA distribution according to time and interaction type. Names and abbreviations of 26 quantified AA are presented in Table A1. (**c**) Score plot on AA content data from a 2+2+0 OPLS-DA model (R^2^_(cum)_ = 0.995, Q^2^_(cum)_ = 0.967) using interaction type as categorical variable. (**d**) OPLS-DA-derived biplot on AA content data using interaction type as categorical variable. (**e**) VIP values from previous OPLS-DA model.

During the time course, the most abundant amino acids (AA) in extracts of *P. indica* (among 26 quantified AA, see Table A1, Figure 3a) were Gln, followed by Ala, Arg, and Glu. However, interactions with soil bacteria caused some variations in the fungal free AA contents (see Figure 3a). Co-culture with *A. chroococcum* caused an increase of Ala, whereas the amounts of Gln and Glu were considerably reduced in comparison to the control. In contrast, Pro, Ile, Val, and Leu showed an important increase, which was up to 4.0, 4.7, 4.2, and 4.6-fold, respectively, when compared to the control. Other AA showed no substantial changes (Figure 3b). Co-culture of *P. indica* with *Streptomyces* AcH 1003 affected the fungal AA pattern in a different way. In contrast to *A. chroococcum* and the control, glutamine was notably increased by AcH 1003. In addition, Gly and Ser were increased drastically. Their accumulation was up to 2.0 and 2.2-fold compared to the control, whereas Cys and cystathionine/methionine showed a meaningful reduction. The other amino acids presented no important variations (Figure 3b).

The orthogonal partial least squares discriminant analysis (OPLS-DA) model (performed on AA content data using interaction type as supervision variable) clearly discriminated *P. indica*–*A. chroococcum* and *P. indica*–*S. anulatus* interactions, according to the respective score plot (Figure 3c) compared to the control. This model involved a well-explained variance (R^2^_(cum)_ = 0.995) using a 2+2+0 component system. This indicates that the observed differences among interactions are due to a remarkable change in AA contents. The corresponding biplot (Figure 3d) revealed the most-influencing AA for the resulting discrimination. Thus, *A. chroococcum* was related to the changes to Ala, GABA, Pro, Lys, and others, while interaction with *S. anulatus* induced the Gln and Ser accumulation in *P. indica*. Some amino acids (such as Thr, Cys/Met, among others) were related to the control in the OPLS-DA model because these had higher concentrations/contents in control, but no substantial changes in concentrations were observed on interaction experiments. For this model, major influences were consequently found to be for Gln and Ala, followed by GABA, Asn, and Leu, according to the variable influence on projection (VIP) values (Figure 3e).

**Figure 4:**
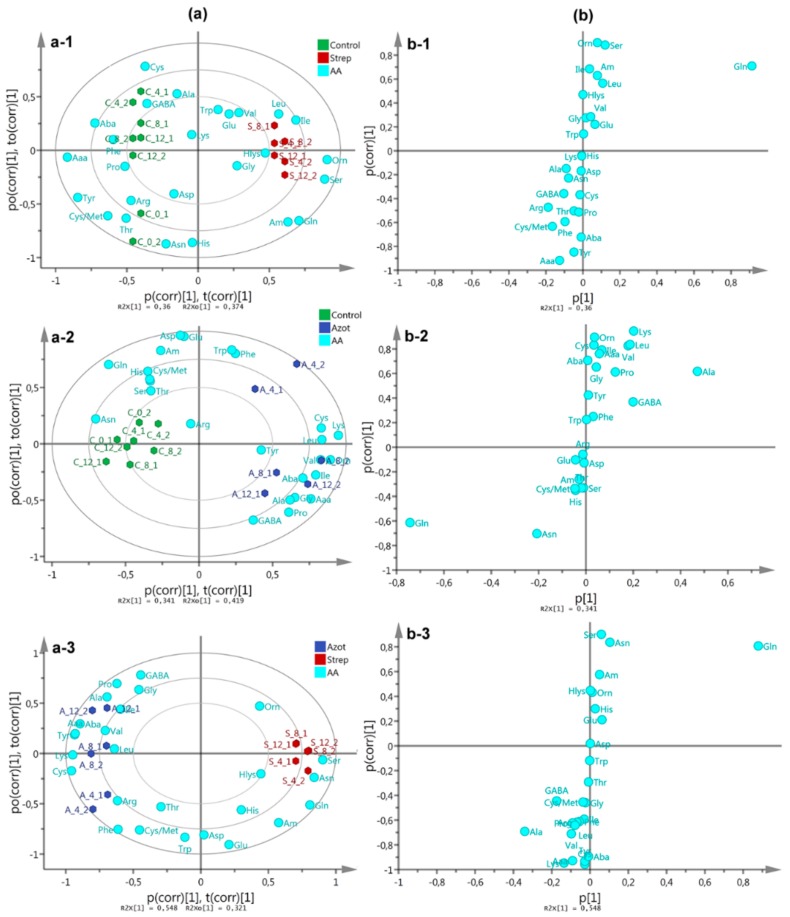
(**a**) OPLS-DA-derived biplot on AA content data using interaction type as categorical variable per each dual comparison: (a-1) Control vs. *S. anulatus* 1003, (a-2) Control vs. *A. chroococcum*, (a-3) *S. anulatus* 1003 vs. *A. chroococcum*, (**b**) OPLS-DA-derived *S*-plot from previous OPLS-DA model: (b-1) Control vs. *S. anulatus* 1003, (b-2) Control vs. *A. chroococcum*, (b-3) *S. anulatus* 1003 vs. *A. chroococcum*.

OPLS-DA models for dual comparisons were also tested. The resulting biplots (Figure 4a) showed good separations along the first principal component (PC1), but interaction time discriminated some observations along the first orthogonal component (OC1). In this regard, the AA content evolved particularly different for the fungus-*A. chroococcum* interaction compared to that with *S. anulatus*. Therefore, AA content was particularly altered depending on the interaction, but the most-affected AA content was also different. Respective *S*-plots (Figure 4b) specified the most affected AA for each comparison. *S. anulatus* interaction led to a Gln accumulation, whereas Ala was induced by the interaction with *A. chroococcum* (Figure 4a-3). The control also exhibited the accumulation of Gln, but the respective concentration of Gln in experiments testing the fungus–bacteria interaction was found to be altered.

**Figure 5:**
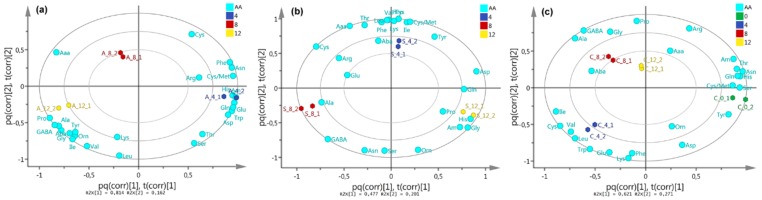
OPLS-DA-derived biplots on evolution of AA content through interaction time in days (as categorical variable) per interaction: (**a**) *A. chroococcum*, (**b**) *S. anulatus*, (**c**) control.

OPLS-DA models using interaction time as supervision variable allowed identifying that time-dependent evolution of these contents was also found to be an important factor for the AA metabolism since interactions during three different periods of incubation were statistically discriminated. In the case of *A. chroococcum* (Figure 5a), the most important AA changes occurred between 4 and 12 days of interaction, and the levels of Aaa caused a different clustering along PC2 at eight days, although also influenced the 12-days cluster along PC1. In addition, the change in Gln for *A. chroococcrum* was more related to four days in combination with Arg and Asn (and other 10 AA), while Ala, GABA, and Leu (and other 6 AA) were accumulated in higher amounts after 12 days. In contrast, *S. anulatus* caused an accumulation of Gln (as well as His, Gly, Am, Pro, and also Asp) at 12 days, Ala and GABA were more affected after eight days, and Leu and Val (and other 9 AA) after four days. In both interactions with *A. chroococcum* and *S. anulatus* 1003, Cys/Met and Phe levels influenced the discrimination at four days (Figure 5b). Cys, Arg, and Glu influenced differentially the discrimination of four and eight days, since four-days cluster was influenced by these AA along PC2, whereas eight-days cluster was marked along PC1, being this influence more important due to the explained variance of PC1 (47.7%). The controls showed a fluctuation in relation to incubation time. In the control, 8 AA (such as Tyr, Cys/Met, Ser, His, Gln, Asn, Thr, and Am) are mainly accumulated at the beginning (0 day) whereas Ala, Gly, Aba, and GABA are mostly produced/accumulated after eight days of incubation (Figure 3c).

**Figure 6:**
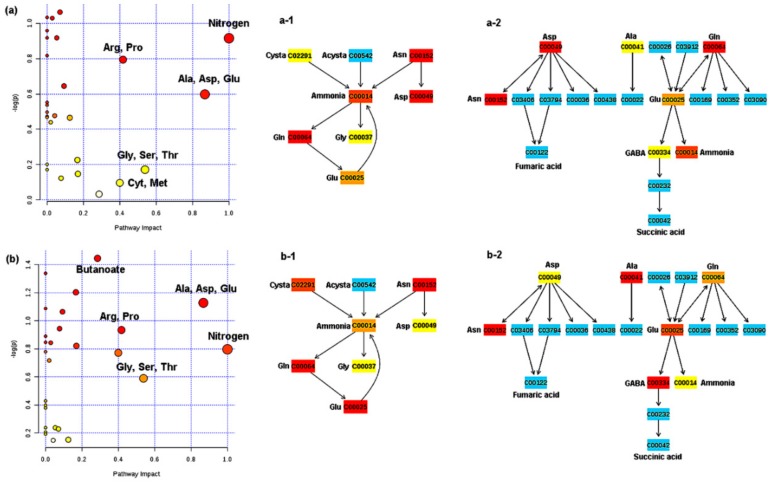
Pathway nodes for (**a**) *P. indica*-*A. chroococcum* and (**b**) *P. indica*-*S. anulatus* interactions. Matched pathways were obtained according to *p* and pathway impact values from PEA and PTA algorithms, respectively, using a pathway library of a model microorganism. (1) Nitrogen metabolic pathway and (2) Ala, Asp, and Glu metabolism as the most important matched pathways, according to the pathway library within the KEGG database. Compound colors within the pathway: Light blue (metabolite not included in current analysis but used as background for PEA), from yellow to red (metabolites included in current analysis with different significance levels, yellow least, red most significant.

In order to rationalize alterations of the AA content of *P. indica* in DC with soil bacteria, a pathway enrichment analysis (PEA) and a pathway topology analysis (PTA) were also performed using the model KEGG library associated to the Pathway Analysis module within Metaboanalyst 3.0. The overview of this pathway analysis (Figure 6) revealed the impact of the *P. indica*-bacterial interactions in some fungal AA metabolism. The two interactions showed different pathway impact profiles, but they exhibited nitrogen and Ala-Asp-Glu metabolism as the most importantly impacted pathway nodes. However, these nodes were found to be opposing in the Pathway Analysis according to their *p* values. Thus, *A. chroococcum* seems to affect: (1) The nitrogen metabolism in *P. indica* in general (Figure 6a-1), or (2) the Ala, Asp, and Glu metabolism (Figure 6a-2) specifically by altering the stored amounts of Asp and Gln as products or precursors, respectively, of these pathways. On the other hand, *S. anulatus* AcH 1003 induces accumulation of Asn and Glu within nitrogen metabolism in general (Figure 6b-1), and/or Ala, Asn, and GABA for the specific pathway (Figure 6b-2).

## 3. Discussion

### 3.1. Effect of the Soil Bacteria on the Growth of P. indica

The establishment of the symbiosis between beneficial endophytic root fungi and plant roots is affected by other microorganisms in the rhizosphere, especially by bacteria [7]. Interactions between soil bacteria and symbiotic fungi can have a positive, negative, or no effect on fungal growth [19]. In the present study, the effect of different soil bacteria on the growth of *P. indica* was analyzed using a fungus–bacterium DC system that has been used before for in vitro studies to elucidate the effect of mycorrhiza helper bacteria on the growth of mycorrhizal fungi and mycorrhiza formation [10]. Results obtained support former studies by Pham et al. [16] which revealed the stimulation of the growth of the fungus *P. indica* by the rhizobacterium *Azotobacter chroococcum* and showed the inhibition of fungal growth through the *Pseudomonas* strains *P. putida* and *P. fluorescens*. In addition, in the present work, the effect of different streptomycete strains isolated by Maier et al. [10] on the growth of *P. indica* was analyzed. Here, AcH 1003 and 2009 inhibited fungal growth, whereas AcH 505 had no effect. The interaction between the bacteria and *P. indica* resulted in non-isodiametric fungal colonies, which indicates that the bacterial exudates influencing the hyphal growth had a slow diffusion rate in the culture media.

Two bacterial strains with a contrasting influence on the development of *P. indica* were selected for biochemical analysis. Interaction between the fungus and *A. chroococcum* resulted in a considerable increase in fungal growth rate (as presented in Figure 2). This positive impact on *P. indica* growth was accompanied by an enhancing of the hyphal extension (Figure 2e) with delayed sporulation, but no effect on hyphal diameter was observed (Figure 2d). Similar results were reported recently by Bhuyan et al. [20] for the co-culture of the *P. indica* with the *Azotobacter* strain WR5 on the same Kaefer agar medium, showing a highly significant growth stimulation in comparison with other *Azotobacter* strains. *A. chroococcum* is a well-known free-living, *N*-fixing rhizobacterium, which is capable of improving plant growth through nitrogen fixation or by the production of growth-promoting substances [21]. The ability to fix nitrogen has also drawn interest to the use of *A. chroococcum* as bioinoculant to increase crop yield and productivity. Recently, the synergistic effect of dual inoculation of *A. chroococcum* with *P. indica* or arbuscular mycorrhizal fungi (AMF) on *Panicum miliaceum* [22], *Stevia rebaudiana* [23], Apple [24], and *Artemisia annua* [25] has been studied. The beneficial effects of MHB are attributed to the production of diverse metabolites. There are several reports stating that *Azotobacter* produces siderophores and indole acetic acid (IAA). These compounds may exert pronounced effects on plant growth by acting as mycorrhizal growth-promotors, as well as facilitators of phosphate solubilization and uptake [26].

In contrast, a fungistatic effect of AcH 1003 on the growth rate of *P. indica* during dual culture was observed. In combination with a reduction of the colony growth of *P. indica* (as observed in Figure 2a), this *Streptomyces* strain also had a notable effect on the fungal development by affecting the colony morphology (Figure 2e) and increasing the hyphal diameter (Figure 2d), but only when the bacteria and fungus grow in a continuous medium without direct contact. The changes in the hyphal diameter of *P. indica* could be associated with the reorganization of the fungal actin cytoskeleton. The ectomycorrhiza-forming fungus *Amanita muscaria* responded to the streptomycete strain AcH 505 in hyphae with a reduced diameter, which might have been an effect of bacterial substances released into the culture medium [27]. In filamentous fungi, several mutants inducing changes in hyphal morphology have been linked to the reorganization of the actin cytoskeleton [28].

Streptomycetes are known to produce pyrazines, i.e., volatile organic compounds well-known for their antimicrobial activities. These compounds are used as competition tools against other microorganisms that do not reside directly adjacent to each other [29,30]. However, volatiles from AcH 1003 did not show any effects on the mycelial extension rate of the *P. indica.* This suggests that the fungal response to AcH 1003 is generated necessarily by diffusion of bacterial metabolites within the medium, excluding volatile compounds.

Contrary to *P. indica’s* effects, prominent stimulatory effects of streptomycetes AcH 1003 and AcH 505 on *A. muscaria* mycelial growth rates have been reported [10,31]. The shape of fungal colonies remained isodiametric in all cultures, indicating that the growth-promoting compounds excreted by the bacteria were homogenously spread within the medium, and more rapidly diffusing than the mycelial growth rate of the responsive fungus. Such results have been shown for the ectomycorrhizal fungus *Agaricus bisporus* during DC with *Pseudomonas putida*, resulting in an isodiametric colony extension of this fungus [32]. Studies carried out with diverse mycorrhiza fungi showed the ability of *Streptomyces* to improve mycorrhization and mycorrhizal growth [5]. The streptomycetes used in this study also promoted the *Suillus bovinus* growth, whereas a suppressing effect on the hyphal extension of *Hebeloma cylindrosporum* was observed [33]. On the other hand, experiments carried out with streptomycete strains A and H showed growth inhibition of the plant pathogenic fungus *Armillaria* sp. [34]. Similarly, it was demonstrated that AcH 505 suppresses the growth of plant pathogens such as *Heterobasidion annosum* and *Amillaria abietina* [10].

It has been observed that Streptomycetes produce a variety of compounds with a stimulatory or inhibitory activity towards soil microorganisms, Riedlinger et al. [11] isolated and characterized three dominant secondary metabolites from *Streptomyces* AcH 505. They demonstrated that the antibiotics WS-5995 B and C suppressed the growth of *A. muscaria*, whereas the third substance, auxofuran, promoted the hyphal extension significantly. Furthermore, AcH 505 can also trigger the production of effectors in plant-pathogenic fungi [12].

### 3.2. P. indica Amino Acid Content During DC with A. chroococcum and AcH 1003

Free AA represent an important parameter to evaluate the absorption and assimilation of carbon and nitrogen of mycorrhizal fungi [35]. In the present study, substantial changes in the content of some free AA in the fungal mycelia of *P. indica* in DC with the soil bacteria were observed. According to the discriminant analysis in combination with PEA and PTA, the most-impacted pathway nodes were linked to nitrogen and Ala-Asp-Glu metabolism based on the variations of the AA contents (see Figure 6), therefore, it was deduced that bacterial interaction influenced nitrogen-related AA. Thus, during the interaction with *A. chroococcum*, Ala was the most abundant AA whereas glutamine and glutamic acid were considerably reduced (Figure 3 and Figure 4b-2). As *A. chroococcum* can assimilate atmospheric nitrogen and thus produces ammonia, we would have expected an increase of the fungal pool sizes of glutamate and glutamine through glutamine synthetase-glutamate synthase pathway (GS-GOGAT) involved in ammonium assimilation in ectomycorrhizal and endomycorrhizal fungi [36,37]. However, we found a considerable reduction in the concentration of these two amino acids compared to control. Reduction in the concentration of Glu and Gln could be related to the increment in Ala content presumably via alanine aminotransferase (EC 2.6.1.2). Alanine synthesis is an important end-product for the glucose-carbon and a convenient reservoir of both amino groups and pyruvate in mycelia during periods of sufficient nitrogen and carbon supply [35,38]. Ala-derived nitrogen can be converted to glutamate nitrogen with the release of pyruvate, as observed in the ectomycorrhizal ascomycete fungus *Cenococcum geophilum* under carbon starvation [39]. In *Tuber borchii* an increase in the pool size of Ala was also associated with a period of active growth [40]. In a study on differentially expressed proteins of *P. indica* co-cultured with *A. chroococcum* was reported the up-regulation of ENO1 (2-phosphoglycerate dehydratase) and Ure D fungi genes, suggesting that the bacterium could trigger the uptake of hexose sugar by the activation of several glucose transporters. ENO1 is one of the key regulatory enzymes of glycolysis [20]. Ure D is one of the accessory proteins of the apoprotein UreABC which is a nickel-dependent regulatory enzyme involved in recycling of urea [41]. The increase of the Ala content could thus be associated with the growth promotion of *P. indica* during the interaction with *A. chroococcum*.

The decrease in Gln and Glu could be due to either reduced activities of glutamine synthetase (GS) and glutamate dehydrogenase (GDH), or to rapid metabolism of these AA, also explaining the four-fold increase in proline as a typical result of this pathway for cellular homeostasis in fungi [42]. Reduction in the activity of these enzymes in *Laccaria bicolor* has been related to the presence of organic N sources such as alanine or arginine in the culture medium [43]. The excretion of AA into the culture medium has been reported for *A. chrooccocum* [44] and *Beijerinckia derxii* [45], but detailed information on the specific AA released is lacking. Additionally, the ca. four-fold increases of the branched-chain amino acids family (i.e., leucine, isoleucine, and valine) can be then rationalized as a consequence of the competitive carbon flux during interaction.

In contrast, the DC of *P. indica* with *S. anulatus* AcH 1003 caused an increase in the amount of Glu, Gly and Ser in the fungal mycelia (Figure 3 and Figure 4a-2). At the same time, a substantial reduction of *P. indica* growth was observed. Gln, Cys, and Gly are important for the synthesis of the antioxidant, glutathione (GSH; γ-l-glutamyl-l-cysteinyl-glycine), which detoxifies non-enzymatically a series of reactive oxygen species, and is generally present in a high concentration of up to 10 mM in yeasts and filamentous fungi [46]. In yeast, serine *O*-acetyltransferase and *O*-acetylserine sulfhydrylase constitute a pathway for synthesis of cysteine from serine [47]. Subsequently, Cys is converted to methionine via cystathionine and homocysteine [48]. This pathway could explain the increase of Ser as a precursor of Cys and the considerable decrease of methionine and cystathionine by the consumption of Cys for the synthesis of GSH. AcH 1003 produces at least three secondary metabolites, one of which belongs to the group of polyene macrolide antibiotics [49]. These are known to act as fungicides and, together with polyol macrolide antibiotics, have been shown to induce oxidative stress [50]. Some substances produced by AcH 1003 were able to generate oxidative stress which, in turn, results in enhanced production of AA, which are important for GSH generation.

## 4. Materials and Methods

### 4.1. Dual Culture of Piriformospora indica with Soil Microorganisms and Sample Collection

To determine the influence of the bacteria on fungal growth, the mycelium of *Piriformospora indica* was cultivated in 9-cm-Petri dishes on Kaefer [51] agar medium in a DC system, together with the respective soil bacteria. A fungal inoculum with a diameter of 4 mm was excised from actively growing rims of five-day-old fungal colonies, using the wide end of a Pasteur pipette. This was placed onto a plate on a sterile cellophane-sheet (exclusion limit 10 kDa; Folia Wendelstein, Germany). Thus, the fungal inoculum always had the same size and the hyphae could be collected easily at the end of the experiment for further analysis. In the same way, 10 μL of soil bacteria from a liquid culture (OD_600nm_: 0.6–0.7) were inoculated as a line of about 4 cm long directly on the media (outside the cellophane sheet) at 4 cm from the fungal colony. The Petri dishes were incubated in darkness at 25 ± 2 °C. In order to select two antagonistic bacterial strains, one that inhibited and one that promoted the fungal growth, dual cultures of *P. indica* with *Azotobacter chroococcum*, *Pseudomonas fluorescens*, and *Pseudomonas putida* were carried out. The latter were isolated and kindly provided by Prof. A. Varma, School Life Science, New Delhi, India. In addition, we investigated the effects of *Streptomyces anulatus* (Beijerinck) Waksman 1003 (AcH 1003), *Streptomyces* sp. nov. 505 (AcH 505), and *Streptomyces* 2009 (AcH 2009). These bacteria were isolated from the hyphosphere of *Picea abies* [10]. In this experiment, ten replicates were used. The effect of the fungus-bacteria interaction on fungal growth was evaluated after twelve days as the radial growth of the colony portion that was oriented towards the bacteria. From this assay, two bacterial strains were selected, *A. chroococcum* as growth-promoting bacterium and *Streptomyces* AcH 1003 as a growth-inhibiting bacterium. Cultures of *P. indica* with *A. chroococcum*, AcH 1003, and fungus alone as control were carried out using the same DC system. Samples of mycelia from the first centimeter of the proximal part of the fungal colony were collected after 4, 8, and 12 days, rapidly homogenized in liquid nitrogen and freeze-dried for three weeks at below −20 °C. Mycelium from five plates was collected as one replicate, four replicas were obtained for each time point. These samples were used to determine the amino acid content.

### 4.2. Influence of Soil Bacteria on Fungal Growth

The bacterial effect on the growth of *P. indica* in the dual cultures was evaluated every two days. Data were registered as colony area of the part oriented towards the bacteria. At least 10 replicates were used for the growth test.

### 4.3. Morphology of Piriformospora indica Hyphae

The influence of the fungus-bacteria interaction on the thickness of *P. indica* hyphae was determined after 4, 6, 8, 10, and 12 days using a Leica DM500 binocular light microscope (Leica Microsystems GmbH, Wetzlar, Germany). The hyphae thickness was measured approximately 10 μm from the hyphal apex using the program Image J 1.62 (National Institute of Health, Bethesda, MD, USA). At least 100 replicates per time point from independent colonies were used. Additionally, the mycelium morphology after 12 days of interaction was registered by optical micrographs at 100× using a Leica S9 D stereo microscope (Leica Microsystems GmbH, Wetzlar, Germany).

### 4.4. Soil Bacteria Culture

Twenty mL of each soil bacteria strain were cultivated in liquid Kaefer media (1977) in a 100 mL-Erlenmeyer flask, containing one metal spiral. *A. chroococcum, P. putida,* and *P. fluorescens* were initially incubated for two days, whereas *Streptomyces*-strains were incubated for four days. The cultures were grown at 28 °C on a rotary shaker at 180 rpm. New cultures were started inoculating 2–4 mL of the cultures incubated for four days in 20 mL of liquid Kaefer media until OD_600nm_: 0.6–0.7 was reached. These cultures were used to inoculate the plates of the dual cultures. The stocks of bacteria were grown at 28 °C in solid Kaefer medium and maintained at 4 °C in the cold room. The stock cultures were replicated every six weeks.

### 4.5. Determination of Amino Acid Content

The determination of the content of 26 different amino acids (Table A1) in mycelia of *P. indica* was carried out as follows: 7 to 10 mg of freeze-dried homogenized mycelia were re-suspended in 500 μL of 80% (*v*/*v*) methanol and heated in a water bath for 3 min. After centrifugation 10 min at 16,069 g, the supernatant was placed on ice and the pellet was resuspended with 500 μL of methanol 20% (*v*/*v*) and heated for 3 min in a water bath. The cell debris was removed by centrifugation at 16,069 g, and the supernatant was transferred into an Eppendorf tube, together with the first extract. The extract was dried in a speed vac centrifuge and the pellet resuspended in a lithium buffer (Li 220 Pickering Laboratories, Mountain View, CA, USA). Analysis was by high performance liquid chromatography (HPLC) on a cation-exchange column (high efficiency fluid column, 3 × 150 mm; Pickering Laboratories, Mountain View, CA, USA) using lithium buffer as eluent. Amino acids were derivatized with ninhydrine before photometric detection as described by Pilot et al. [52]. Three replicates were measured at each time point. The results were reported as nmol amino acid/mg dry weight (DW).

### 4.6. Data Analysis

Differences in the amino acid content between treatments in response to the effect of the fungal bacteria interaction were analyzed with a one-factor ANOVA followed by Tukey’s studentized range (HSD) test using the SAS 8.0 (SAS Institute, Cary, NC, USA).

Targeted metabolomics was also implemented to analyze the amino acid concentrations data under supervised analysis according to fungal bacteria interaction and time. Thus, the resulting two-way array raw data were organized, and a 20 observations (treatments) × 26 variables (amino acid contents in nmol/mg DW) matrix was built. OPLS-DA models were then constructed for classification according to the holistic variation of amino acid content between treatments. OPLS-DA models were created using SIMCA 14 software (Umetrics Inc., Umeå, Sweden) on a PC (Core i5 processor; 8 GB RAM; on Microsoft Windows 7). Integrating enrichment and pathway topology analyses were performed according to the comprehensive, Web-based tool MetaboAnalyst 4.0 (McGill University, Montreal, QC, Canada).

## 5. Conclusions

Fungal growth and its amino acid composition during the interaction of *P. indica* and soil bacteria were studied. Fungal growth was stimulated by *Azotobacter chroococcum*, whereas *Streptomyces anulatus* AcH 1003 inhibited it and *Streptomyces* sp. Nov AcH 505 had no effect. OPLS-DA model clearly discriminated *P. indica*-*A. chroococcum* and *P. indica*-*S. anulatus* interactions. The most-observable responses were found to be in the cases of glutamine and alanine size groups. In this sense, while *Streptomyces* AcH 1003 increased the amount of glutamine, *A. chroococcum* decreased it. The fungal growth and the increase of alanine content might be associated with the assimilation of nitrogen in the presence of glucose as a carbon source. Hence, the *N*-fixing bacterium *A. chroococcum* should stimulate fungal amino acid metabolism via glutamine synthetase-glutamate synthase (GS-GOGAT). The data pointed to a stimulated glycolytic activity in the fungus observed by the accumulation of alanine, possibly via alanine aminotransferase. Responses toward the growth-inhibiting *Streptomyces* AcH 1003 suggested an oxidative-mediated stress response of the fungus. However, additional evidence should be compiled to support it, for instance, by means of the determination of ROS or lipids peroxidation products in fungal cells. Additionally, an important limitation of this AA metabolism study, as a phenotypic response of *P. indica* facing the contrasting bacterial interactions, is related to the direct assignment of the role of each AA. In this regard, these results can be combined with gene expression in order to explain such roles at the biochemical level. However, the present results defined an important parameter to describe a functional bacteria–fungus association to be used properly in symbiotic-mediated plant nutrition.

## Appendix A

**Table A1:** Name and abbreviations of quantified amino acids for *P. indica* in control and dual cultures (bacterial interactions).

No	Name	Abbreviation	No	Name	Abbreviation
**1**	glycine	Gly	**14**	hydroxylysine	Hlys
**2**	serine	Ser	**15**	glutamic acid	Glu
**3**	cysteine	Cys	**16**	glutamine	Gln
**4**	ornithine	Orn	**17**	alanine	Ala
**5**	histidine	His	**18**	arginine	Arg
**6**	tyrosine	Tyr	**19**	asparagine	Asn
**7**	isoleucine	Ile	**20**	proline	Pro
**8**	threonine	Thr	**21**	valine	Val
**9**	α-aminobutyric acid	Aba	**22**	leucine	Leu
**10**	cystathionine/methionine ^a^	Cys/Met	**23**	lysine	Lys
**11**	Phenylalanine	Phe	**24**	ammonia	Am
**12**	aminoapidic acid	Aaa	**25**	aspartic acid	Asp
**13**	Tryptophan	Trp	**26**	γ-aminobutyric acid	Gaba

^a^ cystathionine and methionine could not be separated since they were part of the same chromatographic signal. Hence, the amounts could not be individually determined.
